# Design for Pandemic Information: Examining the Effect of Graphs on Anxiety and Social Distancing Intentions in the COVID-19

**DOI:** 10.3389/fpubh.2022.800789

**Published:** 2022-05-18

**Authors:** Jing Luo, Yaqi Zhang, Yao Song

**Affiliations:** ^1^Department of Industrial Design, College of Art and Design, Shenzhen University, Shenzhen, China; ^2^School of Design, The Hong Kong Polytechnic University, Hong Kong, China; ^3^Department of Advertising, College of Literature and Journalism, Sichuan University, Chengdu, China; ^4^Digital Convergence Laboratory of Chinese Cultural Inheritance and Global Communication, Sichuan University, Chengdu, China

**Keywords:** graph, culture, anxiety, social distancing, data visualization, COVID-19

## Abstract

To increase public awareness and disseminate health information, the WHO and health departments worldwide have been visualizing the latest statistics on the spread of COVID-19 to increase awareness and thus reduce its spread. Within various sources, graphs are frequently used to illustrate COVID-19 datasets. Limited research has provided insights into the effect of different graphs on emotional stress and ineffective behavioral strategies from a cross-cultural perspective. The result of current research suggests a graph with a high proportion size of the colored area (e.g., stacked area graph) might increase people's anxiety and social distancing intentions; people in collectivist culture might have a high level of anxiety and social distancing intentions; the effect of different graphs on social distancing intentions is mediated by anxiety experienced. Theoretical contribution and practical implications on health communication were also discussed in this study.

## Introduction

In January 2020, the World Health Organization (WHO) announced the outbreak of the new coronavirus disease COVID-19 as a public health emergency of international concern and that the risk of COVID-19 spreading worldwide was high (1–4). In March 2020, COVID-19 was further listed as a pandemic. Though the latest medical technology has dramatically promoted health conditions (1, 5), it is still necessary to increase public awareness and disseminate health information. Indeed, the WHO and health departments worldwide have been visualizing the latest statistics on the spread of COVID-19 to increase awareness and thus reduce its spread (6).

Individuals are exposed to information on COVID-19 daily through media such as newspapers, television, and the internet from sources such as the WHO Coronavirus Disease Dashboard (6), Worldometer dashboard (7), or Johns Hopkins Corona Virus Resource Center (8). Within various sources, graphs are frequently used to illustrate COVID-19 datasets. The more commonly used graphs are line, bar, and stacked area graphs (9). However, individuals experience mental stress when processing risk information (10). The difference in the presentation of charts or graphics affects individuals' perceptions and behavioral intentions (11). For example, research has suggested a positive relationship between seeking coronavirus updates and anxiety, common mental stress (12). However, limited research has provided insights into the effect of different graphs on emotional reactions in the context of a pandemic. Considering that social distancing might play an indispensable role in controlling and suppressing the spread of COVID-19 (13), this study aims to investigate the effects of the most commonly used types of graphs (line graphs, bar graphs, and stacked area graphs) on individuals' mental health and preventive behaviors from a cross-cultural perspective.

## Background

### Graphs in Data Visualization

In 1786, William Playfair first introduced graphs to visualize data (14). Since then, an increasing number of researchers have attempted to adopt this graphical representation tool and explore its potential advantages and disadvantages among different graphs (15). As a critical statistical representation tool, graphs have been used as a critical visual communication solution in various fields, such as science, technology, business, education, and mass media (16).

However, graph design guidelines and their effect on viewers have largely been neglected in the literature: individuals have generally relied on their intuition or common sense to make a “good” graph, although this is not always “scientific” (17). Indeed, graphs are more attractive than numbers because they are visually stimulating and can be perceived in a quick, automatic manner, despite some graphs required extra cognitive effort to make estimations (18). For example, the interpretation and calculation of particular graphics may depend on cognitive processing (19). Thus, an ideal graph should be designed to exploit visual heuristics while decreasing cognitive load (20).

Graphs are also widely adopted in printed and electronic materials among various health areas, such as risk assessment, risk signaling, and risk communication (18). However, scant literature has discussed how individuals interpret these public health graphs and how the associated perceptions raised. The understanding of graphs is usually different from what the designer planned. Accordingly, it might be both theoretically and practically significant to investigate the particular visual effects of public health communication on individuals' mental health and related behavior.

### Graphs, Anxiety, and Social Distancing

We reviewed the daily COVID-19 updates from official resources, such as WHO, and observed different types of graphs. When visualizing COVID-19 updates, the most common information resources frequently use two types of graphs: thematic mapping and time series graph (6). For COVID-19 updates, a thematic map shows the spatial distribution of confirmed cases or deaths for selected geographic areas, and a time-series graph is used, for example, to visualize trends of total or daily confirmed cases or deaths over a period of time.

**Figure 1:**
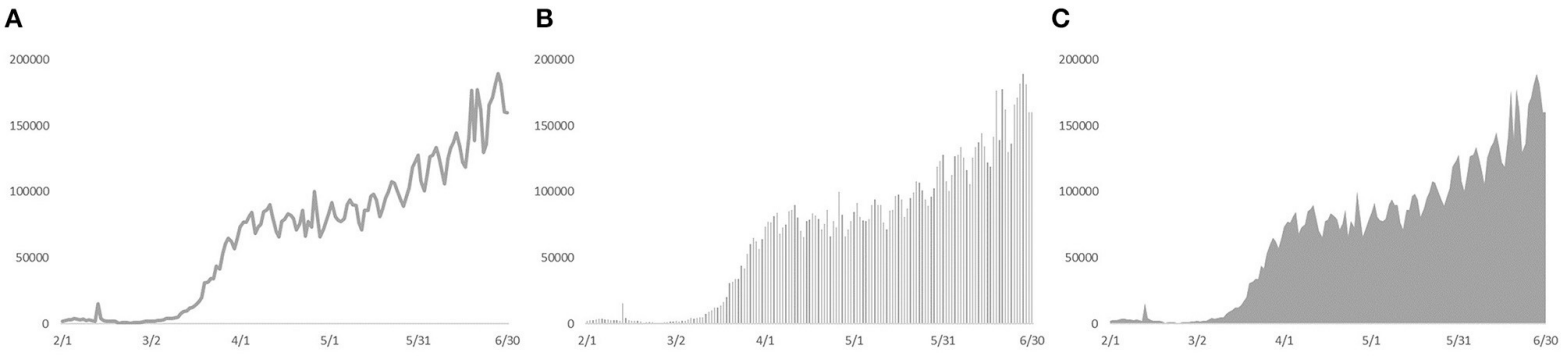
Graphs of daily confirmed cases from Feb 1 to Jun 30, 2020. **(A)** refers to a line graph; **(B)** refers to a bar graph; **(C)** refers to a stacked area graph.

Within time-series graphs, there are three common graphs used to visualize COVID-19 updates: line, bar, and stacked area graphs (Figure 1). Figure 1 illustrates trends of the global total of confirmed cases from Feb 1 to Jun 30, 2020: Figure 1A is a line graph that displays cases as a series of data points connected by straight line segments; Figure 1B is a bar graph that presents data by using rectangular bars with heights or lengths proportional to the cases per day; Figure 1C is a stacked area graph that uses the area between the axis and a line to graphically display data.

Visually, the most significant difference among these three graphs is the different portion sizes of foreground colored area on the background-colored area. In Figure 1, the foreground color of the stacked area graph is gray, and its background-color is white; thus, it has the highest portion size of the colored area, followed by the bar and line graphs. When individuals observe the health updates graph, its information could be perceived as threatening: dissemination of pandemic information might not only improve public awareness but also potentially dampen social wellbeing, such as people's anxiety about the crisis (21). The reason might lie in the bias in the embodied cognition where numerical estimation did not follow a linear relationship with spatial estimation (22). To specify, people tend to overestimate the associated number for high-intensive space while underestimating the number for low-intensive space (23). Thus, people would like to have a stronger sense and overestimate for a large area (24–26). Considering portion size has been demonstrated to have a significant positive impact on anxiety (27, 28), a reasonable prediction is that a graph with a large portion-size area, compared with a small area, could cause a higher level of anxiety in the context of COVID-19 updates.

In addition, many studies have shown that individuals' perceptions of risk and public awareness are positively correlated with higher intentions for ineffective behavioral strategies (5, 29, 30). Accordingly, anxiety might mediate the effect of different graphs on intentions for social distancing (31).

### Cultural Difference in Anxiety and Social Distancing

The research has also suggested that the anxiety of specific populations may be affected by their personality traits, such as cultural elements. According to social identity theory, collectivist self-esteem refers to individuals' self-esteem in relation to the social network to which they belong, rather than respect for themselves (32). Specifically, collectivist self-esteem indicates the extent to which individuals evaluate social groups (33). Compared with individualistic cultures, such as the Caucasian culture in the United States (US), collectivist self-esteem plays a more significant role in collectivist cultures such as China (34). That is, individualistic cultures value the expression and proposition of individual desires, and collectivist cultures pay more attention to maintaining group harmony (34).

Collectivist self-esteem also has a significant impact on the mental health of individuals compared with individualistic cultural backgrounds (35). For example, residents of East Asian countries (36) and immigrants from East Asia to Western countries (37) tend to experience higher levels of social anxiety than individuals from individualistic cultures since collectivism requires people to feel their obligations and responsibilities to group members (38). When exposed to risk information, such as COVID-19 updates, collectivists have stronger emotional reactions and experience higher pressure than individualists (39, 40) because they might have a stronger intention to avoid the risk and return to normal, maintaining group harmony (41). Therefore, an expectation is that in the context of visualizing COVID-19 updates, stacked area graphs might aggravate the anxiety perception by collectivist people (42). In other words, it might be that graph types and cultures could jointly influence anxiety perception and social distancing intentions.

## Study Hypothesis

Based on the literature shown above, hypotheses (H1a to H3b) are stated as follows:

**H1a**: A graph with a high (vs. low) proportion size of the colored area might result in a high (vs. low) level of anxiety.
**H1b**: Individuals in collectivist (vs. individualistic) cultures tend to experience a high (vs. low) level of anxiety.
**H2a**: A graph with a high (vs. low) proportion size of the colored area might have high (vs. low) social distancing intentions.
**H2b**: Individuals in collectivist (vs. individualistic) cultures tend to have high (vs. low) social distancing intentions.
**H3a**: Perceived anxiety mediates the effect of different graphs on social distancing intentions.
**H3b**: Perceived anxiety mediates the effect of different cultures on social distancing intentions.
**H4**: Graph types and cultures jointly influence anxiety and social distancing intentions.

## Experiment Design

**Figure 2:**
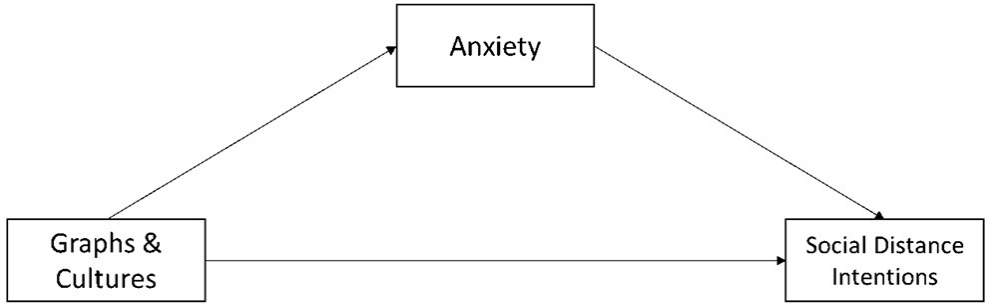
Theoretical model of this study.

The experiment was designed to examine the main effect of different graphs and cultures on social distancing intentions and the mediating role of anxiety in this process (Figure 2).

### Participants and Design

A three (different graphs: line graph, bar graph, and stacked area graph) by two (different cultures: individualism vs. collectivism) between-participants experiment was conducted. Specifically, participants in this experiment were from two sources: the Chinese sample and the US sample. The Chinese sample was recruited from Wenjuanxing (43), and the US sample was recruited from Amazon Mechanical Turk (AMT) (44). Both Wenjuanxing and AMT are crowdsourcing platforms to recruit individuals and conduct behavioral research because of their adequate reliability and validity (16, 45, 46). We posit that both platforms are good data collection sources, especially considering their widely distributed population, which can avoid sampling bias to a large extent (16, 45, 46).

### Stimuli/Material

This study was approved by the Ethical Committee of Shenzhen University (SZUDA20190901001). In the selection of an appropriate index to describe the trend of the COVID-19 crisis, total confirmed cases, daily confirmed cases, and deaths are the most common indicators (6). Nevertheless, these three indicators are of different magnitudes: total confirmed cases and deaths are cumulative time series data, and daily confirmed cases are daily time-series data, which vary frequently. On one hand, it might be inappropriate to introduce three indicators in one graph due to their significantly different magnitudes. On the other hand, total confirmed cases or deaths (total cumulative count) might focus more on the overall severity of the pandemic, while daily new cases might work as a more obvious indicator for trends and predictions regarding COVID-19 (47). Thus, we choose daily new cases as an indicator in this study. Because we investigated the role of cultural differences in response to different graphs, daily confirmed cases for the specific country might bias individuals' emotional status (48). Accordingly, we focused on the global COVID-19 daily new cases, rather than a particular country. To sum up, COVID-19 daily new cases (from Feb 1 to Jun 30, 2020) were extracted from the WHO data repository to illustrate the trends for COVID-19. Microsoft Excel, as one of the most data visualization tools, was used to visualize the COVID-19 data into three graphs (see Figure 1).

### Measurement

As for measurements, since the graph of daily new cases is efficient to illustrate the latest trends of COVID-19, it might influence participants' current anxiety level and their anticipation of COVID-19. Thus, current anxiety level and anticipated anxiety level were measured separately by using the participants' responses to two items on a nine-point Likert scale (My current level of anxiety about COVID-19 is high; If I were to develop flu-like symptoms tomorrow, I would be anxious) (31). Since current anxiety level and anticipated anxiety level constituted the participants' overall anxiety level, overall anxiety was treated as the main factor and measured as the average of current anxiety and anticipated anxiety. In addition, social distancing intentions were measured with a single item (I will avoid going to crowded places in the next few days) on a nine-point rating scale (31). Cronbach's alphas of overall anxiety (0.73) were checked and achieved adequate reliability (49), suggesting it was appropriate for further analysis.

### Sample Size Justification

Power analysis for ANVOA was performed to determine the sample size per group (50). Software G^*^Power was performed on the effect size of Cohen's d = 0.40 and results showed 30 participants per group in the current setting was adequate to attain 80% power (alpha was set at 0.05) (51). Thus, one hundred and eighty participants were recruited in the experiment (mean age = 33.43, SD = 10.47; 107 males and 73 females; 90 US participants and 90 Chinese participants; 14 participants with high school or below, 75 participants with some college, 91 participants with college graduate or above).

As for the analysis plan, the effect of cultures and graphs on current anxiety, anticipated anxiety, and overall anxiety was firstly explored with descriptive analysis, examining H1a and H1b. Then, the effect of cultures and graphs on social distancing intentions was further analyzed, testing H2a and H2b. In addition, the mediating analysis of anxiety in signaling social distancing intentions was conducted to confirm H3a and H3b. Further, to examine H4, we tested the interaction effect of cultures and graphs on anxiety and social distancing intentions. Last, a chi-square test was analyzed to investigate whether people would have a bias for different graphs. The significance level in the analysis was set at 0.05.

### Procedure and Statistical Analysis

The procedure of this experiment involved four parts: pre-study, recruiting, introduction, and the main study. We initially recruited 60 participants *via* Wenjuanxing and conducted a pre-study to ensure the clarity and consistency of the questionnaires. The formal recruitment was followed up after confirmation of the appropriateness of the questionnaire design. Specifically, the experimental task was distributed *via* two channels: participants were recruited with the help of Wenjuanxing for collectivist culture and AMT for individualistic culture. After informing their unique ID will be recorded for research purposes, participants who consented to be enrolled in this study could click the checkbox, “I agree to participate in the research”, and proceeded. For the main study, they were first asked to provide demographic information and were then randomly assigned to one of three stimuli (each graph was seen by 30 participants). Subsequently, they were asked to pay attention to the specific COVID-19 graph for 5 s (52), and then, were instructed to answer a set of questions for anxiety evaluation. Last, they were instructed to estimate the daily confirmed cases on Jun 30, 2020, from four choices (A. approximately 140,000 per day; B. approximately 150,000 per day; C. approximately 160,000 per day; D. approximately 170,000 per day). According to the data from the WHO, 159,962 individuals were confirmed on Jun 30. Thus, approximately 160,000 per day (C) was the correct choice. After finishing all the questions, they were informed they have finished the work. SPSS software was used to perform statistical analysis.

## Data Analysis and Results

**Figure 3:**
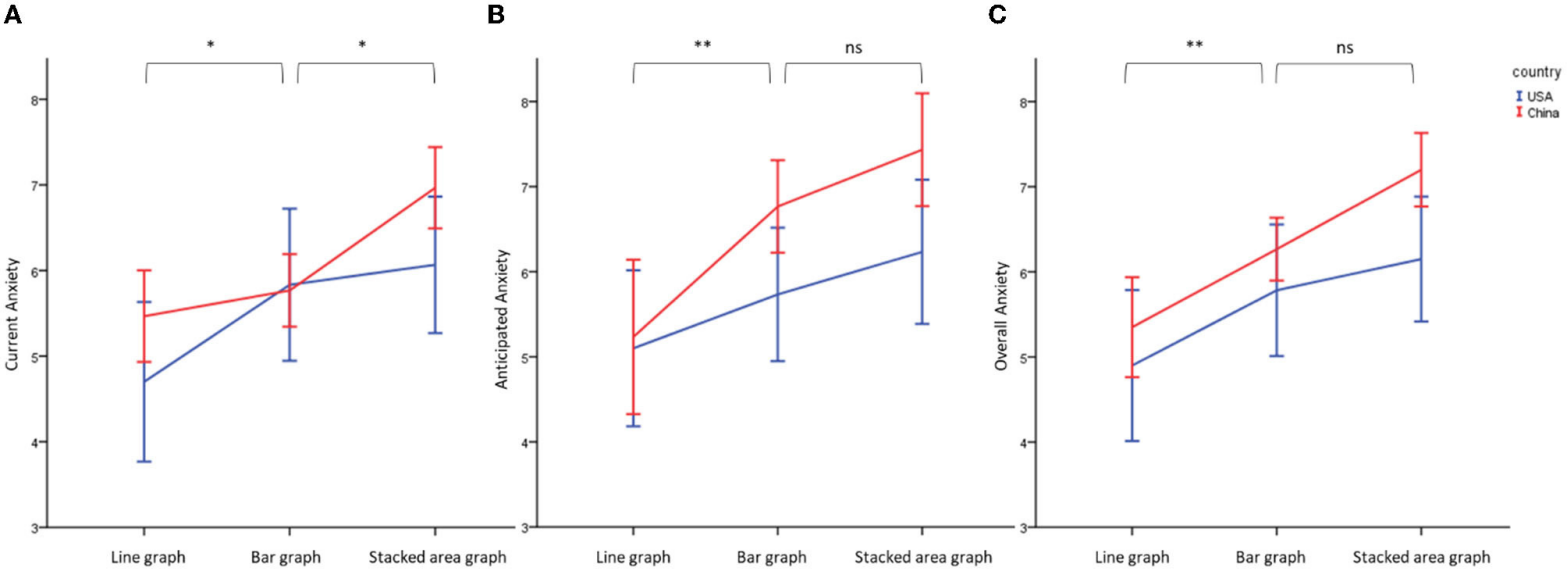
Effect of different graphs and cultures on current anxiety **(A)**, anticipated anxiety **(B)**, and overall anxiety **(C)**. **Significant at 0.05; * Significant at 0.10, ns, non-significant.

To test for H1a and H1b, a two-way ANOVA was conducted with different graphs (line graph, bar graph, and stacked area graph) and different cultures (individualism vs. collectivism) as the independent variables, and current anxiety, anticipated anxiety, and overall anxiety level as the dependent variables. Supporting H1a, the results showed the significant main effect of different graphs: individuals exposed to the stacked area graph had the highest level of current anxiety [Mean = 6.52, SD = 1.79; *F*_(2, 174)_ = 8.64, *p* < 0.01, Eta-squared = 0.09], anticipated anxiety [Mean = 6.83, SD = 2.10; *F*_(2, 174)_ = 9.62, *p* < 0.01, Eta-squared = 0.10], and overall anxiety [Mean = 6.68, SD = 1.68; *F*_(2, 174)_ = 11.73, *p* < 0.01, Eta-squared = 0.12]; the bar graph had a medium level of current anxiety (Mean = 5.80, SD = 1.84), anticipated anxiety (Mean = 6.25, SD = 1.86), and overall anxiety (Mean = 6.02, SD = 1.62); and the line graph had the lowest level of current anxiety (Mean = 5.08, SD = 2.05), anticipated anxiety (Mean = 5.17, SD = 2.42), and overall anxiety (Mean = 5.13, SD = 2.01). Further *post hoc* tests are illustrated in Figure 3.

Partically supporting H1b, the results also indicated the marginally significant main effect of culture (significant for the anticipated and overall anxiety while non-significant for the current anxiety): individuals in China have a higher level of current anxiety [Mean = 6.07 vs. 5.53, SD = 1.42 vs. 2.39; *F*_(1, 174)_ = 3.59, *p* = 0.06, Eta-squared = 0.02], anticipated anxiety [Mean = 6.48 vs. 5.69, SD = 2.12 vs. 2.30; *F*_(1, 174)_ = 6.27, *p* = 0.01, Eta-squared = 0.04], and overall anxiety [Mean = 6.27 vs. 5.61, SD = 1.46 vs. 2.18; *F*_(1, 174)_ = 6.35, *p* = 0.01, Eta-squared = 0.04] than US individuals. However, there is no significant interaction effects on current anxiety [*F*_(2, 174)_ = 1.15, *p* = 0.31], anticipated anxiety [*F*_(2, 174)_ = 1.10, *p* = 0.33], and overall anxiety [*F*_(2, 174)_ = 0.55, *p* = 0.57].

**Figure 4:**
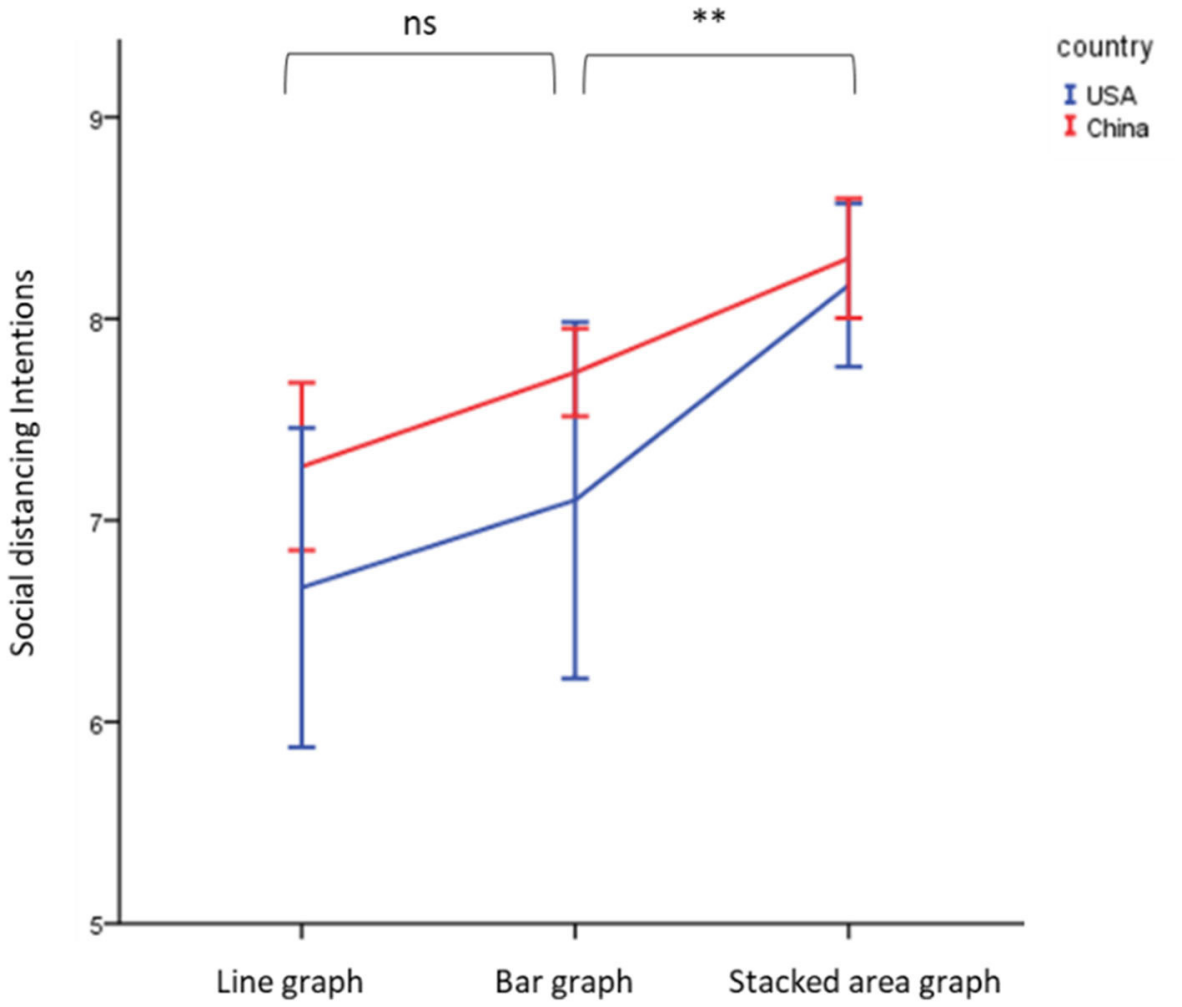
Effect of different graphs and cultures on social distancing intentions. **Significant at 0.05; ns, non-significant.

Similarly, the two-way ANOVA was also performed on social distancing intentions. The results showed that individuals exposed to the stacked area graph experienced a higher social distancing intentions [Mean = 8.23, SD = 0.94; *F*_(2, 174)_ = 10.99, *p* < 0.01, Eta-squared = 0.12] than for the bar graph (Mean = 7.42, SD = 1.74) and line graph (Mean = 6.97, SD = 1.70), and the difference in social distancing intentions between Chinese and US individuals were significant [Mean = 7.77 vs. 7.31, SD = 0.94 vs. 2.02; *F*_(1, 174)_ = 4.15, *p* = 0.04, Eta-squared = 0.02]. Thus, H2a and H2b was supported (Figure 4). However, there was no significant interaction effect between different cultures and graphs [*F*_(2, 174)_ = 0.52, p = 0.59]. H4 was not supported.

**Figure 5:**
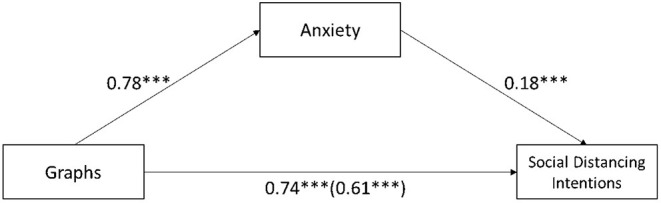
Mediation analysis of anxiety between different graphs and social distancing intentions. ***Significant at 0.01.

**Figure 6:**
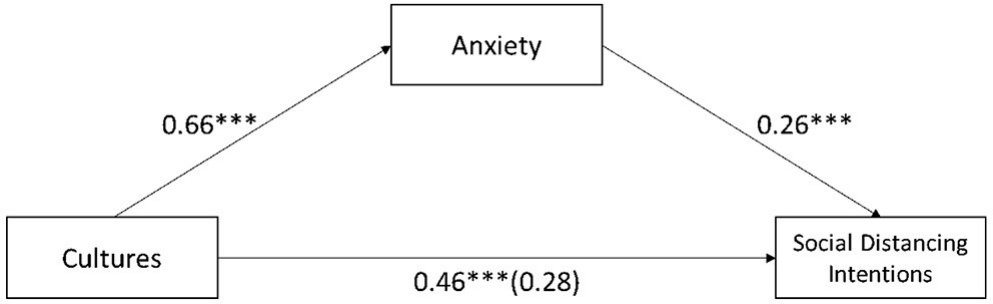
Mediation analysis of anxiety between different cultures and social distancing intentions. ***Significant at 0.01.

To test H3a and H3b, the mediating role of anxiety and social distancing intentions were regressed on different graphs and cultures with overall anxiety as the mediator (model 4; *n* = 5,000 resamples; Hayes, 2015). According to the results, we observe a significant mediation effect for graphs (β = 0.16, SE = 0.08, LLCI = 0.02, ULCI = 0.20) and cultures (β = 0.17, SE = 0.09, LLCI = 0.03, ULCI = 0.37), separately. Figures 5, 6 show the separate mediation analysis.

**Figure 7:**
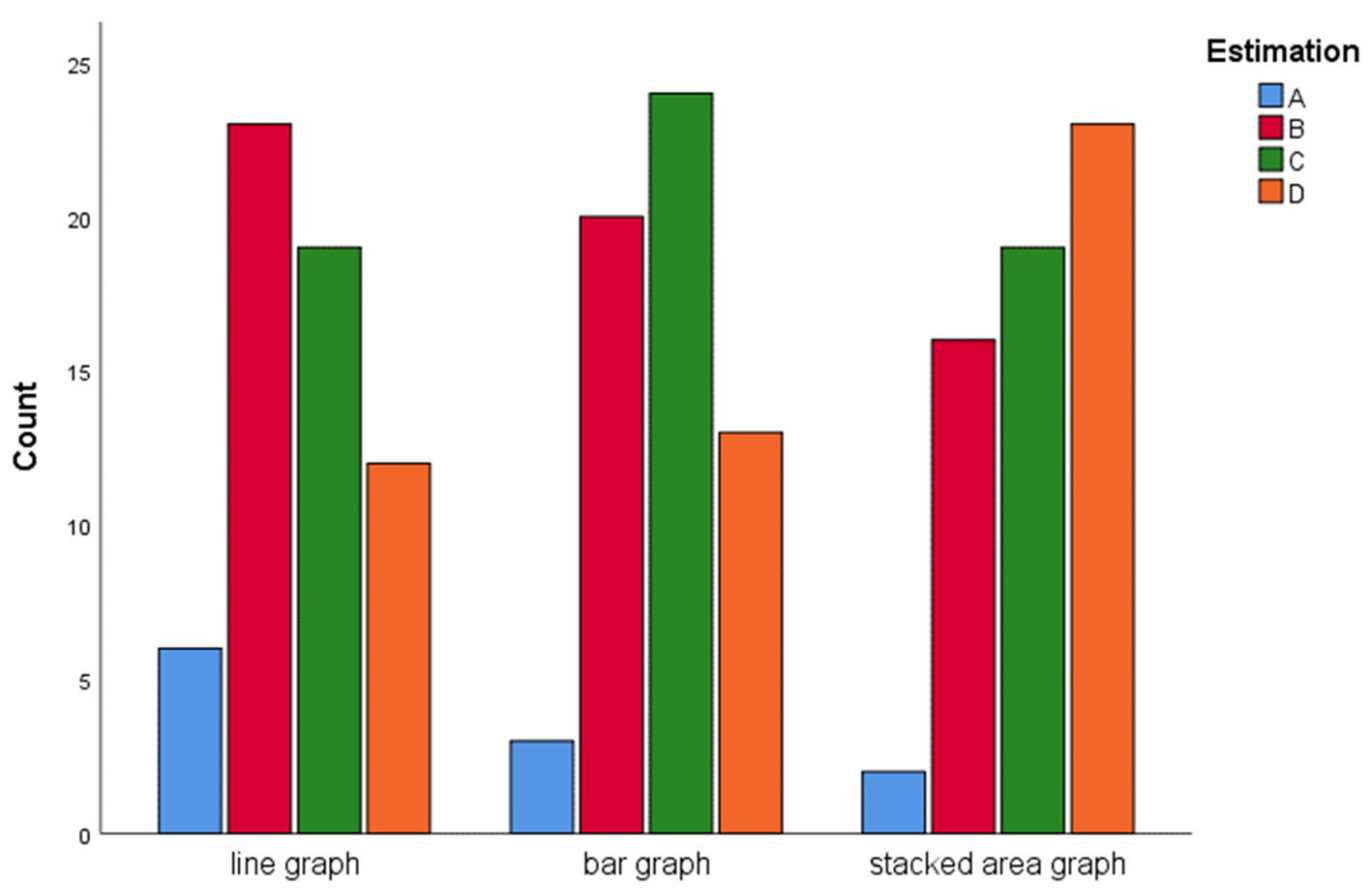
Estimation counts of the new cases on Jun 30, 2020.

Last, for new case estimation, we observe that the participants in the stacked area graph tended to overestimate the daily cases (the most common response was option “D”), and individuals exposed to the line graph underestimated the daily cases (the most common responses were options “A” and “B”). A chi-square test (6, *N* = 180) = 9.04, *p* = 0.17, showed no significant difference between different graphs and different options (Figure 7).

## Discussion

### Theoretical and Practical Implications

This study mainly makes two theoretical contributions to the literature. To begin with, this study contributes to data visualization theory in the context of COVID-19. The rapid speed of the information and communications technology evolution has fundamentally changed how individuals obtain the latest public health information (53). Indeed, an increasing number of individuals are searching for health-related information on the internet (54). Regarding the intuitive nature of the visual presentation, graphs on health information have been widely used (55). Research on health information visualization has mainly focused on two perspectives: the difference between graphs and numbers (e.g., a table), and different design features within one graph (e.g., the size of a graphic element) (18). Scant research has attempted to discuss the most common types of graphs and their impact on health information communication. Thus, it is theoretically significant to examine the relationship between different graphs and their influences on behavior or behavioral intentions. Consistent with the research on embodied cognition (23, 27, 28), the stacked area graph resulted in a higher level of anxiety and social distancing intentions, followed by the bar graph and line graph. Although a stacked area graph might work as a better visualization tool to improve public awareness, it still could increase people's anxiety and dampen social wellbeing. Accordingly, we might still face an ethical dilemma: stacked area graphs might help to communicate health information and decrease people's intentions to get crowded while it could also threaten some residents who have already experienced stress, anxiety, and even depression *via* various social media (56). Related authorities might need to strike a balance between increasing public awareness and its potential deficiency and implement necessary mental services, such as a hotline, to support public mental health, especially vulnerable people (57). Another intriguing thing is through a line graph might not promote public awareness and social distancing, individuals might still prefer and like it for its simplicity and familiarity (58).

In addition, the current study validates the finding that, compared with individualism cultures in Western countries, individuals living in collectivist cultures, such as that in China, might generally experience higher social anxiety (59). However, the cultural elements did not aggravate the anxiety perception and social distancing intentions for collectivists, namely, different cultures might similarly proportional influence the effect of graphs on anxiety perceptions. Moreover, regarding the effect size of cultures and graph types, results showed the effects of cultures on anxiety and social distancing intentions were relatively small (<0.06) while the effect of graph types was relatively medium (>0.06) (60). It might suggest that graph types might have a more significant impact on anxiety and social distancing, compared with different cultures.

As for social distancing intentions, we observed a significant difference between different cultures, which is consistent with previous research on cultural differences in social distancing: people from individualistic countries are less likely to commit the social distancing as the governmental suggestions (61). Accordingly, there seem tradeoffs between the degree of freedom and constraints from the various viewpoints across societies when committing social distancing (61).

This study also has the following managerial implications. First, because the outbreak of COVID-19 continues to pose significant challenges worldwide (6), it is critical to increase public awareness and encourage social distancing (62). Compared with line graphs and bar graphs, stacked area graphs might work as an efficient tool to increase public health awareness and social distancing toward the COVID-19 crisis, nevertheless, we cannot neglect its potential threats on social mental health and should further consider specific characteristics of the audience. For example, since the whole world is still currently within the COVID-19 pandemic, different countries or regions might face different outbreak stages of COVID-19. Thus, some people in a collectivist culture might generally experience a higher level of anxiety when exposed to the same health information. Considering striking a balance between public wellbeing and social distancing, it might be more appropriate to use different graphs for different residents based on their current situations.

### Limitations and Further Research Directions

This study has limitations that require further investigations. First, although the line, bar, and stacked area graphs are the most common time-series graphs in communicating COVID-19 updates, there are other time-series graphs, such as scatter plots, which are seldom used for health risk communication (9). Thus, further research could comprehensively analyze different time series graphs' influence on public health awareness and risk-prevention behavior. In addition, the graphic proportion of different graphs is the main means for communicating risk-related information; however, there are many other design elements in graph design, such as color, font size, and animation, which might elicit public health awareness and social distancing (18). For example, the colors red and yellow could significantly elicit higher anxiety than the colors blue and green (63) because higher levels of Chroma might increase greater feelings of excitement and more intense behavioral reactions (64). Regarding the WHO COVID-19 dashboard's general use of the colors gray or blue in visualizing data (6), the effect of graph color and its interaction with different graph types on public health awareness and social distancing requires further research to determine the most appropriate combinations of graph design elements. Further, though social distancing intentions could be influenced by social anxiety, they might also be shaped by various factors, such as local restrictions and governmental regulations (65) and resident characteristics (66). Thus, a possibility that different cities within one country might have different social distancing policies, such as the limitation of public gatherings to four persons in Hong Kong (67), which might elicit individuals' social distancing intentions to some extent (65). Last, for the measurements of anxiety, we used a single-item scale (or the average of two items) to assess the related anxiety level. Although a single-item scale might enjoy relatively similar reliability and validity compared with the multiple-item scale, the multiple-item scale indeed outperformed the single-item scale in specific cases (68). A further study should consider local regulations and examines their impact on people's social distancing intentions and applied a multiple-item measurement of related anxiety to validate the current finding.

## Conclusion

Considering COVID-19 information visualization is widely used in various media communications of the latest health updates, this study examined the effect of different graphs and cultures on individuals' anxiety levels and social distancing intentions. Specifically, we indicated the mediation effect of anxiety on the relationship between different graphs and social distancing intentions and the role of different cultures in responding to different graphs. The results of this study demonstrate the following: (1) the stacked area graph caused the highest level of anxiety and social distancing intentions, followed by the bar and then line graphs; (2) Collectivist residents tended to experience a higher level of anxiety and social distancing intentions than individualistic residents; (3) there were no interaction effects of different graphs and cultures on anxiety level and social distancing intentions; (4) the effect of different graphs and cultures on social distancing intentions was mediated by anxiety level; (5) individuals exposed to the stacked area graph tended to overestimate the daily confirmed cases, and those exposed to the line graph tended to underestimate the daily confirmed cases.

## Data Availability Statement

The raw data supporting the conclusions of this article will be made available by the authors, without undue reservation.

## Ethics Statement

The studies involving human participants were reviewed and approved by the Ethical Committee of Shenzhen University (SZUDA20190901001). The patients/participants provided their written informed consent to participate in this study.

## Author Contributions

JL, YZ, and YS: conceptualization, methodology, and writing—original draft preparation. YS: formal analysis. YZ and JL: investigation. JL: resources. JL and YS: data curation. All authors contributed to the article and approved the submitted version.

## Funding

This research was funded by Shenzhen University, “The Fundamental Research Funds for the Central Universities” [YJ202203], Annual Program of Social Science Research of Sichuan Province for the Thirteenth Five-year Plan [SC20ZT006], and The Innovation Project of Shanghai Municipal Education Commission [2017-01-07-00-06-E00031].

## Conflict of Interest

The authors declare that the research was conducted in the absence of any commercial or financial relationships that could be construed as a potential conflict of interest.

## Publisher's Note

All claims expressed in this article are solely those of the authors and do not necessarily represent those of their affiliated organizations, or those of the publisher, the editors and the reviewers. Any product that may be evaluated in this article, or claim that may be made by its manufacturer, is not guaranteed or endorsed by the publisher.
