# Supplementary figures and images for: Design for Pandemic Information: Examining the Effect of Graphs on Anxiety and Social Distancing Intentions in the COVID-19
Source: Front Public Health. 2022 May 18;10:800789. doi: 10.3389/fpubh.2022.800789 (PMC9158495; doi:10.3389/fpubh.2022.800789)

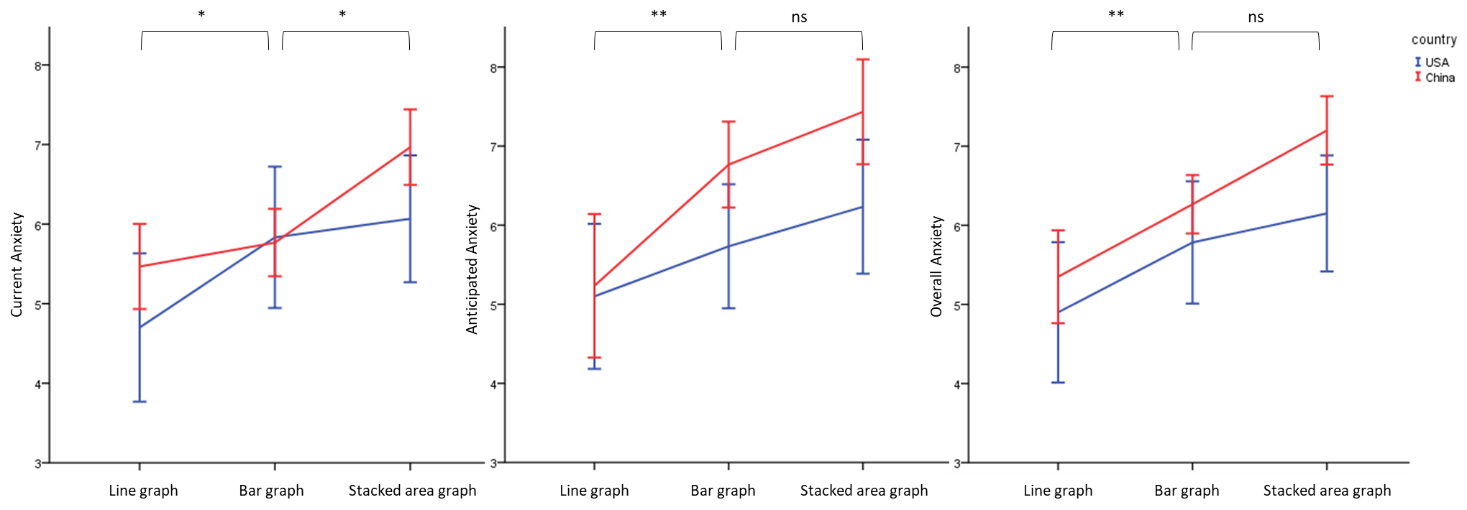

Supplement: Supplementary file 2 [file Image_1.TIF]

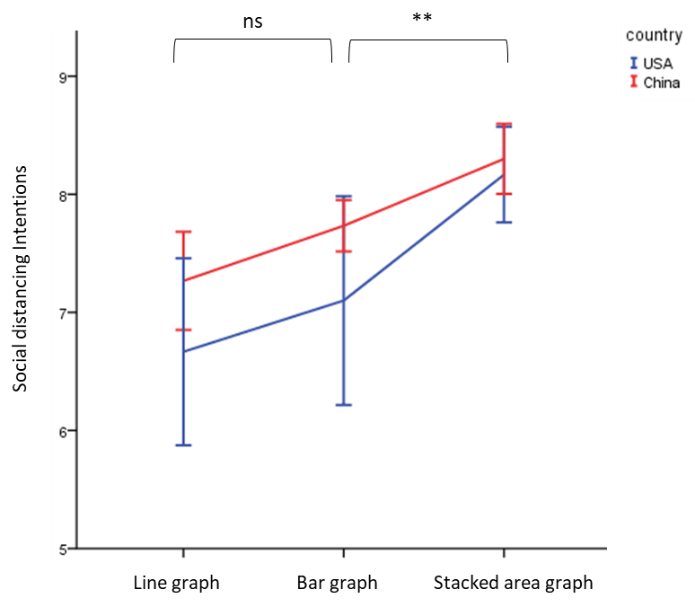

Supplement: Supplementary file 3 [file Image_2.TIF]
